# Bacteriophage-Based Biotechnological Applications

**DOI:** 10.3390/v11080737

**Published:** 2019-08-10

**Authors:** Sílvio B. Santos, Joana Azeredo

**Affiliations:** Centre of Biological Engineering, University of Minho, Campus de Gualtar, 4710-057 Braga, Portugal

## Abstract

Phages have shown a high biotechnological potential with numerous applications. The advent of high-resolution microscopy techniques aligned with omic and molecular tools are revealing innovative phage features and enabling new processes that can be further exploited for biotechnological applications in a wide variety of fields. This special issue is a collection of original and review articles focusing on the most recent advances in phage-based biotechnology with applications for human benefit.

## 1. Introduction

The global threat of antibiotic resistance is dramatic and is driving the rebirth of bacteriophage research—the study of viruses that specifically infect bacteria. Such research is resulting in an increasing wealth of knowledge on phages themselves and also on their genes and proteins, something that is being fostered by the recent progresses in high-resolution microscopy, sequencing technologies, DNA manipulation, and synthetic biology approaches with all the molecular tools that are now available. This knowledge is revealing more and more features of phages and their proteins, allowing for their further exploitation, either in their natural state or as new and improved engineered forms [1].

Phages have spent billions of years evolving and developing a powerful protein armamentarium to recognize, infect, and kill bacteria in a very efficient way. These phage intrinsic properties enable their exploitation in a variety of different fields, including health, industry, food science and safety, and agriculture, for purposes not limited to bacteria control.

This increasing and innovative research on phages and their proteins is revealing new applications and is now rapidly progressing, something that is reflected in this special issue.

## 2. Applications

### 2.1. Pathogens Control

Phage therapy is gaining an uneven renewed interest due to the recent successful reports on patients’ treatments in Europe and USA as a unique and last therapeutic resource [2,3]. Phage therapy, however, is still controversial due to some concerns related to safety and efficacy. Phage specificity is one advantage of phage therapy in comparison with conventional antimicrobials, however, this specificity may make it difficult to isolate an adequate phage for a particular treatment. To overcome this difficulty, Burrowes and colleagues [4] have studied the “Appelmans protocol”, a protocol that is empirically used by Eastern European researchers to generate therapeutic phages with novel lytic host ranges and which enables the targeting of phage refractory bacteria. The researchers concluded that this simple protocol, that is able to expand the host range of a phage without the addition of new genetic information, works predominantly via recombination between the used phages.

Another challenge faced by the use of phages to control bacteria is the phage stability, and consequently antimicrobial efficacy, under different environmental conditions. Encapsulation, a technique already used for different active compounds to protect them against harsh conditions, was studied by González-Menéndez and colleagues [5] to assess its potential to protect phages under conditions faced during food processing. González-Menéndez and colleagues have shown that, besides allowing the successful encapsulation of phages, nanovesicles maintained the bacteriophage’s infectivity during storage and protected the phage particles from low pH. Encapsulation thus represents an appropriate procedure to protect phages, enabling their application in the food processing industry.

Besides targeting infectious diseases, phages have been proposed as drug delivery vehicles to treat neurogenerative diseases. Philip Serwer and Elena T. Wright [6] have given their opinion on the application of phages in nanomedicine, discussing how phages (more specifically their capsids) can be applied to treat diseases such as Alzheimer’s, Parkinson’s, or even cancer.

Phage endolysins are one of the most promising alternatives to antibiotics due to their enzymatic nature, with the ability to rapidly degrade the bacterial peptidoglycan, causing the death of the cells. In this special issue, we had the contribution of two original articles demonstrating the potential of endolysins on the control of Gram-positive bacteria [7] and of Gram-negative bacteria [8] (for which relatively little is known regarding the spectrum of bactericidal activity). Besides their high potential to control bacteria, knowledge on endolysins’ safety and toxicity profiles is scarce. In this special issue, Marek Harhala and colleagues [9] present preclinical safety and toxicity data for two pneumococcal endolysins.

### 2.2. Pathogens Detection

The inherent phage specificity is fundamental in its interactions with its hosts and depends upon the phage receptor binding proteins (RBPs). These proteins, usually located at the phage tail, are responsible for recognizing specific receptors on the cell surface. The process of host cell recognition and attachment by a bacteriophage remains poorly understood but here, Sergey A. Buth and colleagues [10] shed light on the mechanism of interaction between RBPs and *Pseudomonas aeruginosa* using R-type pyocins as RBPs models. The application of phage RBPs on bacterial diagnosis is then demonstrated by Sonja Kunstmann and colleagues [11], who incorporated these proteins into different detection set-ups to obtain a highly specific and sensitive bacteriophage RBP-based *Shigella* detection system.

The high specificity of endolysins targeting Gram-positive bacteria was shown to be attributed to the existence of a cell-binding domain (CBD). As a consequence, such endolysins’ CBDs can also be harnessed for bacterial diagnosis, as demonstrated by Eunsu Ha and colleagues [7]. The specificity of these domains can also be combined with the specificity of phages themselves by using the first to capture the target cells (using the CBDs to coat magnetic beads) and the second as a bioluminescent reporter to specifically infect those cells and replicate, thus increasing the signal and producing an ultrasensitive and fast diagnostics of viable *Listeria* cells [12].

Another interesting area of research is the application of phages in diagnosis, not to target the bacteria but instead to eliminate common sample contaminations, thus improving the target bacteria recovery and consequently improving detection. Jumpei Uchiyama and colleagues [13] used this approach to remove *Enterococcus faecalis* from vaginal samples, avoiding the overgrowth of these bacteria during the enrichment phase, allowing for the detection of *Streptococcus agalactiae* and decreasing the common high number of false negatives observed with vaginal *S. agalactiae* diagnosis.

### 2.3. Phage Display

The ability of phages to display foreign peptides/proteins on their surface has given rise to the powerful technique called phage display. Typically, this technique allows for the identification of new proteins and peptides with the capability to bind to their target molecules, since it permits a direct linkage between the genotype and phenotype of the phage displaying the peptide/protein of interest. This potentiality was used by Harvinder Talwar and colleagues [14] to identify specific diagnostic biomarkers for the detection of tuberculosis in sera with a high sensitivity and specificity, allowing also for the identification of new mimotopes with applications in therapy and prophylaxis of this disease with global impact.

The advances in structural phage biology and phage display technology have led to the construction of a novel type of phage display library and consequently to new nanomaterials—the landscape phages—with application in different areas of bioscience, medicine, material science, and engineering. Valery A. Petrenko [15] has reviewed the application of these landscape phages, focusing on phage-functionalized biosensors and phage-targeted nanomedicines.

### 2.4. Other Applications

Phages as molecular biology tools have a long past. Nevertheless, new phage genomes and proteomes continue to be exploited for the design of new molecular strategies. A new phage-bacterium system developed by Éva Surányi and colleagues [16] was used as a molecular switch to study protein:DNA and protein:protein interactions in living cells.

In recent years, highly ordered and self-assembly-based nanostructures have generated increasing interest due to their diverse number of applications in different fields of nanobiosciences. Also, phages [17] and their proteins [18] have been found to be useful and show high potential in the construction of such nanostructures.

## 3. Conclusions

The high-quality original articles and reviews present in this special issue demonstrate the incredible potential of phages and derived proteins in a wide range of biotechnological applications for human benefit. We hope that these articles will inspire researchers to investigate new phages, new proteins, and new phage-based processes to solve old and new biotechnological problems.

Considering the arise of amazing new bioengineering tools that are now available and the high abundance of phages and phage-proteins to be discovered and studied, we believe that the next coming years will present us with many more fascinating, new, and previously unthinkable phage-based biotechnological applications.
